# Privacy Preservation in Patient Information Exchange Systems Based on Blockchain: System Design Study

**DOI:** 10.2196/29108

**Published:** 2022-03-22

**Authors:** Sejong ­Lee, Jaehyeon Kim, Yongseok Kwon, Teasung Kim, Sunghyun Cho

**Affiliations:** 1 Department of Computer Science and Engineering Hanyang University Ansan Republic of Korea; 2 Major in Bio-Artificial Intelligence Hanyang University Ansan Republic of Korea; 3 Department of Applied Artificial Intelligence Hanyang University Ansan Republic of Korea

**Keywords:** electronic medical records, consortium blockchain, data security, medical data management, privacy preservation, smart contract, proxy re-encryption, patient-centered medical system, InterPlanetary File System

## Abstract

**Background:**

With the increasing sophistication of the medical industry, various advanced medical services such as medical artificial intelligence, telemedicine, and personalized health care services have emerged. The demand for medical data is also rapidly increasing today because advanced medical services use medical data such as user data and electronic medical records (EMRs) to provide services. As a result, health care institutions and medical practitioners are researching various mechanisms and tools to feed medical data into their systems seamlessly. However, medical data contain sensitive personal information of patients. Therefore, ensuring security while meeting the demand for medical data is a very important problem in the information age for which a solution is required.

**Objective:**

Our goal is to design a blockchain-based decentralized patient information exchange (PIE) system that can safely and efficiently share EMRs. The proposed system preserves patients’ privacy in the EMRs through a medical information exchange process that includes data encryption and access control.

**Methods:**

We propose a blockchain-based EMR-sharing system that allows patients to manage their EMRs scattered across multiple hospitals and share them with other users. Our PIE system protects the patient’s EMR from security threats such as counterfeiting and privacy attacks during data sharing. In addition, it provides scalability by using distributed data-sharing methods to quickly share an EMR, regardless of its size or type. We implemented simulation models using Hyperledger Fabric, an open source blockchain framework.

**Results:**

We performed a simulation of the EMR-sharing process and compared it with previous works on blockchain-based medical systems to check the proposed system’s performance. During the simulation, we found that it takes an average of 0.01014 (SD 0.0028) seconds to download 1 MB of EMR in our proposed PIE system. Moreover, it has been confirmed that data can be freely shared with other users regardless of the size or format of the data to be transmitted through the distributed data-sharing technique using the InterPlanetary File System. We conducted a security analysis to check whether the proposed security mechanism can effectively protect users of the EMR-sharing system from security threats such as data forgery or unauthorized access, and we found that the distributed ledger structure and re-encryption–based data encryption method can effectively protect users’ EMRs from forgery and privacy leak threats and provide data integrity.

**Conclusions:**

Blockchain is a distributed ledger technology that provides data integrity to enable patient-centered health information exchange and access control. PIE systems integrate and manage fragmented patient EMRs through blockchain and protect users from security threats during the data exchange process among users. To increase safety and efficiency in the EMR-sharing process, we used access control using security levels, data encryption based on re-encryption, and a distributed data-sharing scheme.

## Introduction

### Background

With the development of information and communication technology, the existing medical information system, which used paper charts to manage medical information such as patient treatment information and clinical results, changed to a digital-based medical information system. As of 2017, more than 94% of the hospitals in the United States have used digital health information systems [1,2]. The digital medical information system uses electronic medical records (EMRs) that store patient medical information (eg, patient demographics, progress notes, medications, vital signs, past medical history, immunizations, laboratory data, and radiology reports) in electronic document format for patient treatment and health management [3]. Moreover, health care practitioners use EMRs to provide improved health care to their patients through clinical decision support tools [4,5]. Of late, EMRs have been actively used in various fields (eg, medical artificial intelligence development [6-8], clinical trials [8-10], customized health care [11,12], and telemedicine [13,14]) by combining EMRs with the core technologies of the fourth industrial revolution. As EMRs are used widely, the value of, and demand for, EMRs continue to increase, and the size of the medical data market is also increasing every year [15,16]. There have been various attempts to share EMRs using networks to supply scarce EMRs, such as image-sharing networks [17,18] or health information exchange mechanisms [19-21]. However, the existing EMR-sharing systems centered on medical institutions that use a trusted third party (TTP) have security vulnerabilities and structural limitations. In the current EMR-sharing system, which manages data through a central database, overall service can be affected if a problem occurs in the database storing the data. Furthermore, if an attacker forges the EMR, it is difficult to determine whether the EMR has been forged if there are no original data for comparison, and in the case of data loss, permanent loss can occur if there is no backup file to recover the data [22,23]. Moreover, as the EMR-sharing process is performed by a third-party data center or cloud service provider, personal information of the patient can be exposed [24-26]. An EMR contains personal information that can identify the patient. Therefore, privacy issues can arise if sensitive information regarding, for example, abortion clinic visits or records of treatment for sexually transmitted disease, is leaked.

**Figure 1:**
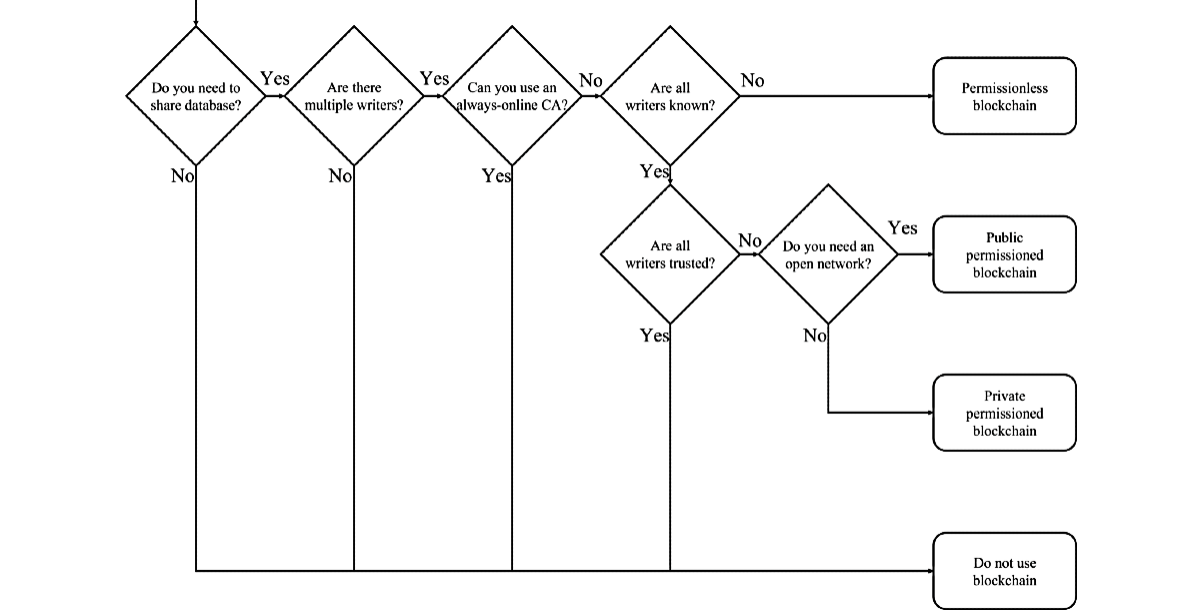
Decision-making flowchart to determine whether blockchain is an appropriate technical solution to a problem, adapted from the study by Wüst and Gervais [38]. CA: certificate authority.

Medical information that directly affects a patient’s health must have integrity and be reliable. Moreover, the patient’s privacy should be protected from exposure to unauthorized users. Therefore, it is necessary to develop a secure EMR-sharing system that can provide the integrity and reliability of an EMR and protect patient privacy by addressing the problems of the existing centralized EMR-sharing systems. Decentralization of the system has been proposed to complement the problems of the existing EMR-sharing system, and blockchain is receiving much attention as a technology suited for this purpose [27,28]. Blockchain stores data using a shared ledger maintained and managed through consensus by nodes participating in a blockchain network. By storing the previous block’s hash value created using an irreversible hash function in the newly created block, blocks form a chain structure in which they are sequentially connected [29,30]. Furthermore, because the data stored on the blockchain cannot be arbitrarily modified or deleted, the blockchain provides strong tamper-resistant performance. Because of these technical characteristics, blockchain provides transparency and integrity of data and enables transactions among users without central administrators and third parties [31]. In addition, the blockchain technology that provides data integrity and transparency through a distributed shared ledger can be made scalable by applying automation technologies such as smart contracts. A smart contract is a digital contract written in code and executed automatically, first devised by Szabo [32]. Since then, smart contracts have been used for digital asset trading on Ethereum, a blockchain platform developed by Buterin [33,34]. By using smart contract technology, users can authenticate the contents of a transaction without the intervention of a third party and can be guaranteed an accurate and automated contract by means of a prewritten code. As blockchain has been applied to various fields, the role of smart contracts has also diversified. When smart contracts are applied to the medical field, various medical services such as remote patient monitoring, clinical trials, and drug supply chain management can be automated [35-37]. Moreover, it is possible to control access rights by using smart contracts so that only users who meet access policies (APs) can access medical data. However, blockchain technology is still at the prototype level, lacks technical stability, and suffers from limited performance, including low throughput and high latency [28,31]. In addition, there are some problems that arise when applying blockchain technology to the EMR system. For example, in the process of propagating transactions to nodes to store data on the blockchain, patient information may be disclosed to multiple users if the EMR is not encrypted. Furthermore, because of the limited block capacity, large-capacity data (eg, medical images) cannot be shared, and there are insufficient measures in place to ensure the patient’s ownership of the EMR. Therefore, to apply blockchain technology to the EMR-sharing system, measures to resolve the aforementioned problems are required. Because of these issues, analysis studies are being conducted on whether it is appropriate to apply blockchain technology to the EMR-sharing system [38]. Figure 1 is a flowchart adapted from the study by Wüst and Gervais [38] to determine whether blockchain is an appropriate solution to the problems of the existing centralized database. To share an EMR, the system needs a shared database. However, if a TTP such as a certificate authority (CA) supervising the entire sharing process is semitrusted, privacy concerns may arise. Therefore, there is a limitation to the use of a TTP for EMR-sharing systems. In our proposed system, there are many patients as well as physicians who write EMRs. They should be identified for the purpose of sharing certain patient information with the appropriate physicians, but they may have concerns about one another’s privacy. Hence, for the EMR-sharing system, permissioned blockchain can be applied.

The blockchain-based decentralized EMR-sharing system has the opposite characteristics to the existing client–server-based centralized system. Through these opposite characteristics, the blockchain-based EMR-sharing system overcomes the current system’s problems and provides various advantages. Unlike the existing centralized system, the blockchain-based decentralized EMR-sharing system exhibits strong resistance to the single point of failure because no central administrator or server controls the system. As multiple nodes operate the decentralized EMR system, data loss or service failure can be prevented even if a specific node fails. Therefore, it is possible to build a more robust system and provide stable service.

In terms of performance, the blockchain-based EMR-sharing system exhibits low throughput and high latency compared with centralized systems because of the data propagation delay between nodes and the consensus mechanism. However, performance problems can be overcome through various methods, including automation of the system by using smart contracts, lightweight consensus mechanisms, and private blockchain models. In a centralized system, only the central administrator manages the database. Hence, the data stored in the database can be arbitrarily modified or deleted only by the central administrator. However, to modify stored data in the decentralized EMR-sharing system, the consent of most of the blockchain nodes is required; therefore, an arbitrary user cannot modify the data at will. Therefore, the blockchain-based EMR-sharing system provides high data integrity and a transparent process, allowing EMRs to be shared without the intervention of a third party, unlike centralized systems. The decentralized EMR-sharing system prevents data leakage and privacy threats from third parties with these characteristics.

Despite many concerns about technological limitations and suitability, many researchers are studying blockchain-based EMR-sharing systems to take advantage of the benefits of blockchain technology [39-42]. Table 1 shows the differences between a blockchain-based distributed EMR-sharing system and a client–server-based centralized EMR-sharing system.

**Table 1:** Comparison of the decentralized (blockchain) and centralized (client–server) electronic medical record–sharing system.

Characteristics	Decentralized system	Centralized system
System-fault tolerance	Strong	Weak
Throughput	Low	High
Latency	High	Low
Data integrity	High	Medium
Trusted third party	No	Yes
Storage	Distributed ledger	Centralized database
Privacy preservation	Strong	Weak

### Related Works: Blockchain Technology in Medical Care Fields

Researchers have proposed various EMR-sharing system models based on blockchain to secure the integrity and reliability of EMRs and build a secure EMR-sharing environment. The studies on EMR-sharing systems based on blockchain technology are presented in Table 2.

Azaria et al [43] proposed MedRec, a decentralized medical record management system based on Ethereum smart contracts. MedRec manages access rights to medical records using smart contracts and permissions stored in the blockchain. When a client sends a query request, the gatekeeper checks the client’s signature and the blockchain contracts to verify access rights. However, because the system proposed by the authors does not encrypt medical data, there is a risk of leakage of personal information and data during the data-sharing process. Besides, when data are shared, the transaction-processing efficiency is degraded because of the additional processing time required as it is necessary for the request queries to be sent from the provider’s local database.

**Table 2:** Blockchain-based electronic medical record (EMR)-sharing systems.

Year	Authors	Description	Limitation	Entities
2016	Azaria et al [43]	The authors proposed a new distributed record management system that handles EMRs Researchers and public health authorities participate in the blockchain network as miners Miners given access to anonymized aggregate data as mining rewards through proof of work	Scalability and security	Patient and provider
2018	Griggs et al [35]	All events between patients and physicians are stored and managed using a customized smart contract in the blockchain All sensor data captured by IoT^a^ devices are stored and managed in the blockchain Smart devices can provide automated alerts using smart contracts to users and health care providers	Scalability and security	Patient and hospital
2018	Uddin et al [44]	Design a lightweight blockchain model and an encryption algorithm for the IoT-based remote patient-monitoring system	Centralization, verification cost, and scalability	Patient, IoT device, cloud service provider, and hospital
2018	Maslove et al [45]	The authors presented a proof-of-concept blockchain-based clinical trial data management solution, enabling patients and researchers to participate in clinical research	Scalability and security	Patient and researcher
2019	Guo et al [46]	The study presents an attribute-based encryption system for authorization and dynamic authentication of medical on-demand services in remote medical systems Data index management using blockchain for data security of public cloud-based telemedicine services	Centralization and security	Patients, hospital, cloud service provider, and authorities
2019	Hylock and Zeng [47]	The authors proposed a proxy re-encryption–based redactable blockchain system for a privacy-preserving and efficient medical data exchange system	Scalability	Patient, hospital, and researcher
2019	Wu and Du [48]	Data-masking techniques were presented to prevent personal information leakage in blockchain-based medical systems IPFS^b^, a distributed file-sharing protocol, was used to share large-capacity data such as medical images	Security	Patient and physician
2020	Abdellatif et al [49]	The authors proposed a system model and priority-based data-sharing algorithm using blockchain and edge computing for remote health care systems	Scalability, security, and centralization	Patient and hospital

^a^IoT: Internet of Things.

^b^IPFS: InterPlanetary File System.

Hylock and Zeng [47] proposed HealthChain to enhance patient engagement and security in blockchain-based health information exchange systems. Proxy re-encryption (PRE) [50-54] technology was used to prevent leakage of patient private keys and medical data. Furthermore, the authors introduced redactable patient blocks with chameleon hashing to solve the data fragmentation problem and reduce storage and computation overhead by modifying the data. However, in the system proposed by the authors, there is a fatal problem: patients must share their private keys with an external third party for re-encryption. Moreover, for patients to share and manage their medical data, they must continuously participate in the blockchain network, burdening patients who have limited resources, unlike hospitals and research institutes.

Wu and Du [48] proposed an EMR security-sharing model based on blockchain for improving privacy and data scalability in medical data–sharing systems. An EMR security-sharing model based on blockchain uses data-masking technology to hide sensitive information stored in the medical data to prevent leakage of personal information. Moreover, the InterPlanetary File System (IPFS) [55], a distributed file-sharing protocol, was used to overcome the difficulty of sharing medical data because of the limited block size. However, depending on the masking level, the privacy protection offered by data-masking technology varies in performance. The problem is that applying excessive masking makes it challenging to use the required information, and when the level of masking is low, specific values can be tracked and predicted.

Abdellatif et al [49] proposed ssHealth, a smart and secure health care system, which is a distributed health care system that enables convenient medical data–sharing among various institutions using blockchain and edge computing. The ssHealth system divides medical data processing, access control, and data sharing into local and blockchain networks and presents a data-sharing security algorithm based on the importance of data to enable safe medical data–sharing. However, there is a problem: it is not possible to guarantee stable service quality because of differences in validation time, depending on the security level. Besides, there is a risk of centralization and privacy breaches because unencrypted medical data and patient personal information pass through edge nodes in the local network.

Existing studies on blockchain-based EMR-sharing systems have used blockchain models designed for cryptocurrencies such as Bitcoin and Ethereum. However, existing blockchain models for cryptocurrency have limitations in providing the security and scalability required in sharing EMRs. The system also failed to meet the requirements of EMR-sharing systems as defined in *Connecting Health and Care for the Nation: A Shared Nationwide Interoperability Roadmap – Version 1.0*, prepared by the Office of the National Coordinator for Health Information Technology (ONC) [56]. Therefore, it is necessary to develop a blockchain-based EMR-sharing system that overcomes the limitations of existing systems and satisfies the security framework defined by the ONC.

In this paper, we propose a patient information exchange (PIE) system. The proposed blockchain-based EMR-sharing system overcomes the limitations of existing blockchain-based EMR-sharing systems and satisfies the privacy and security framework defined by the ONC. Furthermore, our proposed system prevents data loss and privacy breaches in sharing data through the data encryption scheme based on re-encryption, ensuring strong data security. Moreover, data integrity is ensured by preventing the forgery and alteration of EMRs by using the decentralized ledger structure and the unique hash value of the data. Furthermore, allowing patients to set their data-access rights ensures patient ownership of their EMR and establishes a patient-centered medical system. Moreover, the PIE system provides improved performance by solving the low processing performance and scalability issues due to the limited block capacity of the existing blockchain through the distributed data-sharing method using the IPFS. As a result, we contribute business process optimization, cost reduction, patient outcome improvements, and enhanced compliance in the health care field [57,58].

## Methods

### System Model

**Figure 2:**
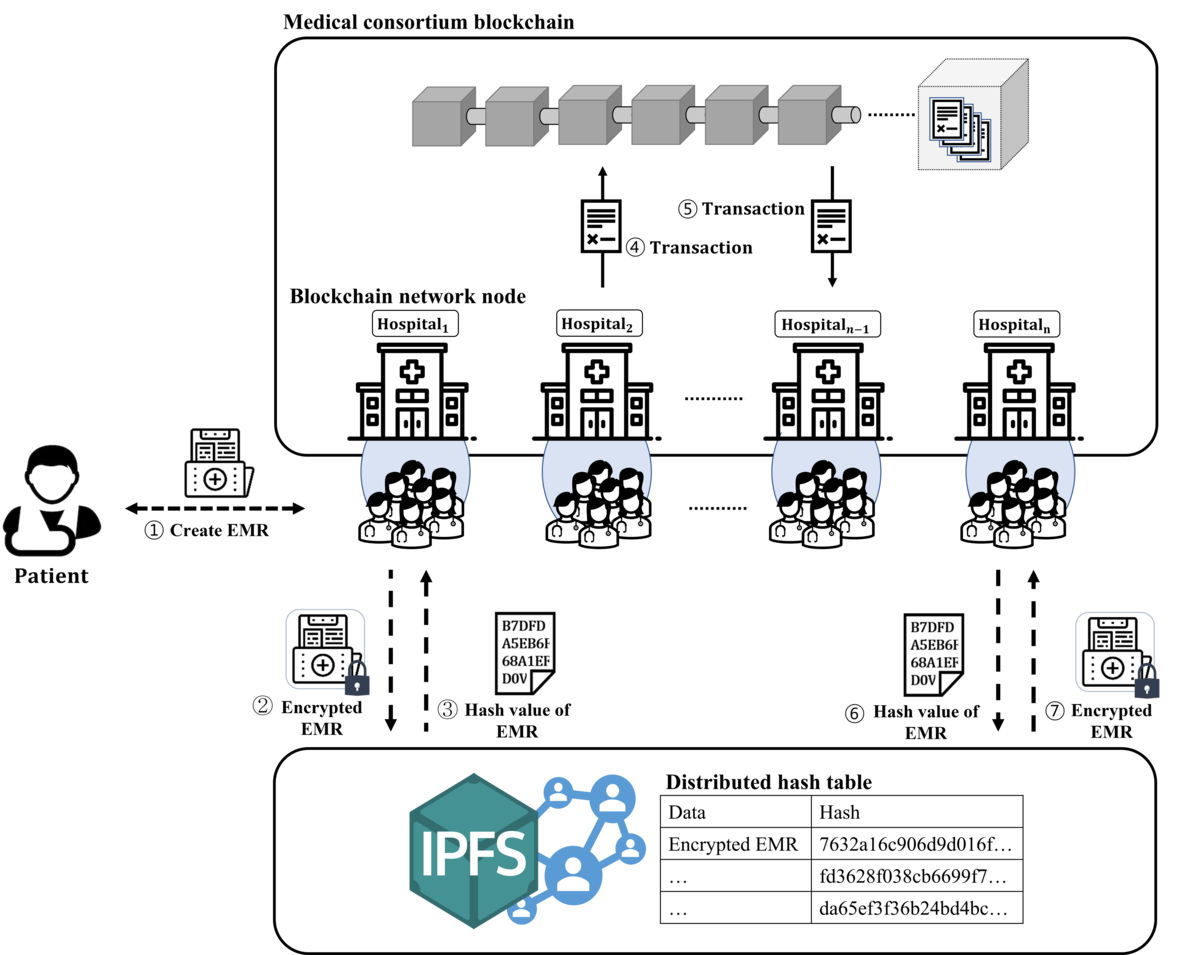
The proposed blockchain-based patient information exchange system model. EMR: electronic medical record. IPFS: InterPlanetary File System.

Here, we describe the proposed PIE system. In the *Components of the Proposed PIE System* section, we define the entities that make up the components of the system and describe each entity’s role. In the *EMR Transaction Structure* section, we describe the structure and components designed to share EMRs effectively. Finally, in the *Security Levels of EMRs* section, we discuss the security level, which depends on the EMR data type and classifies the data based on the type. The system model of the proposed PIE system is shown in Figure 2.

We propose a blockchain-based PIE system to improve the security and efficiency of the EMR-sharing process. To prevent forgery of EMRs and protect patient privacy, we use a consortium blockchain model in which only authorized users can participate. The medical consortium that operates and manages the blockchain comprises state-approved and trusted medical institutions. As the proposed blockchain-based PIE system uses a private blockchain model, the consensus algorithm in the block generation process is not addressed. Instead, the chain is constructed by sequentially storing the generated EMR transactions to create a block and connecting them. Hospitals and medical institutions serve as blockchain nodes that issue EMR transactions and store them in block form. Health care workers and patients who create and use EMRs participate in the blockchain network as users by using IDs issued according to user type after a certification process by a CA. Users participating in the blockchain network can register their EMRs on the blockchain and use them at any time. The proposed PIE system is a patient-centered EMR-sharing system where patients directly participate in the EMR upload and EMR-sharing process. The patient directly generates a key to encrypt the EMR and defines the categories of users who can access the EMR. By allowing patients to manage their own EMRs, we build a user-centered system that protects patients’ privacy and gives them ownership of their EMRs.

The proposed PIE system securely protects patient EMRs from security threats such as data forgery and personal information leakage, which can occur during EMR management and sharing. To protect EMRs from the aforementioned security threats, we use public key–based asymmetric encryption and our proposed PRE-based decryption authority delegation mechanism. The proposed decryption authority delegation mechanism prevents private key leakage by decrypting data encrypted with the public key. Moreover, delegating authority to decrypt data solves the problem of data access in an emergency when a patient cannot respond to a request for access to their EMR, as de Oliveira et al [59] suggested. The proposed PIE system provides a re-encryption key that enables the physician who created the EMR to act on behalf of the patient in an emergency when the patient is unable to control access rights to the EMR. The re-encryption key re-encrypts the EMR encrypted with the patient’s encryption key into a form that the physician can decrypt with the physician’s private key.

The performance and scalability of the PIE system are enhanced by using the IPFS, which supports distributed data-sharing technology. The EMR encrypted with the patient’s encryption key is stored on the IPFS network, and the hash value of the EMR is stored on the medical blockchain in the form of metadata. Instead of storing the data as a whole in the blockchain, it is possible to reduce the load on the system by storing only the hash value of the data. Furthermore, if data are shared using the IPFS, large-capacity data such as magnetic resonance imaging, computed tomography, and endoscopy images can also be shared, improving the scalability of the blockchain system.

### Components of the Proposed PIE System

The proposed PIE system consists of blockchain nodes (medical consortium), users of the blockchain network (patients and health care workers), and the IPFS. The role of each entity is outlined in the following paragraph:

A medical consortium consisting of hospitals and medical institutions that wish to share EMRs builds and manages a distributed ledger as operator of a permissioned blockchain network in which only authorized users can participate. The medical consortium blockchain stores the information of the EMRs generated by each hospital. The information recorded on the blockchain is a hash value of real medical data stored in the IPFS and simplified medical information that users can comprehend. Data registered on the blockchain cannot be arbitrarily deleted or modified, providing high reliability and medical data integrity. Patients and physicians, who are the users of the blockchain network, share EMR information through the network. Patients can use a decentralized app to share their EMRs in the PIE system. Furthermore, patients set their APs for their EMRs and generate re-encryption keys for re-encryption. Unlike traditional hospital-centered health care systems, the PIE system guarantees the patient’s ownership of their EMR. In a patient-centered health care system, where patients have rights to their own EMRs, they have the freedom to choose who can use their EMR and their data at any time. Furthermore, patients may sell their medical data to research institutions or hospitals, in addition to using the data for therapeutic purposes. Health care workers consist of reliable physicians and health care service providers such as medical researchers and insurance agents. Health care workers use computer systems at hospitals or medical institutions to encrypt EMRs generated during the patient treatment process and upload them to the IPFS. After uploading the EMRs, health care workers submit the EMR information to their hospitals and institutions. Health care workers also serve as consumers of medical data by, for example, sharing EMRs through a blockchain network to treat patients or using the data for clinical research. The IPFS is a distributed file-sharing system that splits data stored on multiple computers worldwide into small pieces and shares only a portion. The distributed data-sharing method used by the IPFS enables rapid sharing of large-capacity data such as magnetic resonance imaging or computed tomography images. In addition, the IPFS prevents duplicate creation and storage of medical data by managing data with hash values based on data content.

### Threat Model

In this study, we consider the traditional cryptographic system, not the postquantum cryptographic system. Therefore, we use the discrete logarithm problem, which is one of the difficult problems of 1-way functions. The discrete logarithm problem is one where given x, y ∈ Z^*^_q_, it is difficult for any probabilistic polynomial time attacker A to find a value m ∈ Z^*^_q_ such that *x* = *y^m^*. Therefore, the attacker cannot obtain the private key from a public key or ciphertext. Our system model considers external threats from outside the system and internal threats from the system participants. We assume that both threats are in the form of a logical attack, not a physical attack. The external threats target the patients’ private data such as the EMR, insurance details, and other personal information. For example, an external attacker wants to eavesdrop on all communication among the participants to obtain patients’ personal information. Internal attackers can include health care researchers or insurance agents. They are allowed access to limited information in the form of an abstract regarding disease and length of hospital stay, not details of the disease or patients’ personal information. However, internal attackers are snooping for patients’ private data; therefore, they try to access their medical information. In addition, internal attackers attempt to manipulate clinical results or commit insurance fraud by arbitrarily forging a patient’s EMR. Table 3 shows the attack scenarios and threat situations considered in the proposed blockchain-based EMR-sharing system.

**Table 3:** Attack scenarios and threats considered by the proposed system.

Types and attack scenario		Threats
**External threats**		
	Eavesdropping	Private data leakage (eg, electronic medical record and personal information)
	Denial of service	Service unavailable
**Internal threats**		
	Abnormal access	Private data leakage
	Data forgery	Unexpected output

### EMR Transaction Structure

The proposed blockchain-based medical system uses transactions designed to effectively share the desired medical data while preventing leakage of personal information and data when uploading the medical data to the blockchain. A unique identifier or ID is used in the blockchain network by the physician who created the EMR and the patient who is the owner of the generated data. The CA issues a user ID according to the type of user participating in the blockchain network. A user ID is a randomly generated value consisting of numbers and letters; it is possible to identify users but not know who the owner is. Information about users who can map users to user IDs is securely managed by a CA such as the trusted government authority that issued the ID. As the user IDs are correlated, users are protected from the threat of personal information leakage [60,61]. The timestamps record the time the transaction was created. Medical information contains minimal necessary medical information, excluding sensitive information that can identify the user from the patient’s EMR. For example, even if information such as the gender of the patient, type of disease, age, and exercise status is disclosed, it is not a serious problem because the owner of the data cannot be identified. The information is only used in the search process to identify a specific EMR of interest among the various EMRs stored in the blockchain. The metadata contain the hash value, which is the address value of the data received after uploading the encrypted EMR to the IPFS. Using this, a specific EMR can be shared in the IPFS. The contract code contains the code to execute the smart contract, for example, the user’s AP. The signature is the one created by using the private key of the physician who created the transaction (the physician who treated the patient and generated the EMR). The structure of transactions for effective and safe EMR management and sharing is shown in Table 4.

**Table 4:** Transaction structure for electronic medical record (EMR) sharing.

Field	Definition
User ID	IDs of the patient and physician
Timestamp	Time the transaction was created
EMR information	Summary of information in the EMR
Metadata	Hash value of encrypted EMR
Contract code	Patient’s defined access permission policy
Signature	Signing with the user’s private key

### Security Levels of EMRs

Patient EMRs may include data relating to clinical trials and insurance as well as sensor data generated by health care devices, in addition to medical information generated during the process of receiving treatment in hospitals. Depending on the EMR data type, the required security levels will differ. For example, if information such as name, residence, and social security number, which can identify an individual, is leaked to outside parties, it can lead to serious personal information leakage; consequently, a high security level is required. Conversely, information that is not personally identifiable, such as gender, age, eating habits, and exercise status, does not require a high security level because it is not a serious problem even if this information is disclosed to outside parties. Therefore, it is necessary to provide differentiated security levels and separate management, with respect to the sensitivity of the personal information, according to the EMR data type.

The minimum security level required for each data type is established by categorizing privacy sensitivity according to data type and evaluating accessibility and data potential per user. The security levels assigned according to the sensitivity of private information fall into three classes: private, moderate, and low. Table 5 lists the security levels differentiated according to the type of information contained in the EMR.

**Table 5:** Security levels required depending on the type of information contained in the electronic medical record.

Division and class		Security level
**Medical record**		
	Medical information	Private
	Admission record	Private
	Prescription	Private
	Medical imaging (x-ray, magnetic resonance imaging, and computed tomography)	Private
**Clinical trial**		
	Medical device	Low
	Medicine	Low
	Clinical observation	Low
	Omics (genomics)	Low
**Lifelog**		
	Sensor data (weight, heart rate, and sleep pattern)	Moderate

### EMR-Sharing Process in the PIE System

#### Overview

This section presents the EMR-sharing process that protects the patient’s EMR from various attacks and safely shares it. Moreover, it describes the work performed in each process. A more detailed description of each process-specific algorithm is provided in Multimedia Appendix 1. The proposed EMR-sharing process consists of a user registration phase, an EMR upload phase, and an EMR-sharing phase. The user registration phase concerns joining the blockchain network so that users such as patients and physicians can manage and share medical data. The EMR upload session concerns registering on the blockchain the medical data generated when treating patients. The process of publishing a patient’s EMR on the blockchain involves data encryption, access rights setting, transaction creation, and uploading. Finally, the EMR-sharing phase concerns the process of downloading the encrypted patient EMR from the blockchain, re-encrypting it, and then decrypting it to obtain the original version. The notations used for EMR encryption and re-encryption in the proposed system are listed in Table 6.

**Table 6:** Notations used for electronic medical record (EMR) encryption and re-encryption in the proposed system.

Notation	Description
ID	User ID (patient or physician)
SK	Private key of the user
PK	Public key of the user
DEK	Dedicated encryption key to encrypt EMR
RK	Re-encryption key
C_i_	Encrypted EMR
P_i_	Patient_i_
D_i_	Doctor_i_
AP	Access policy
hash_i_	Hash value of the encrypted EMR

#### Initial Phase: User Registration

**Figure 3:**
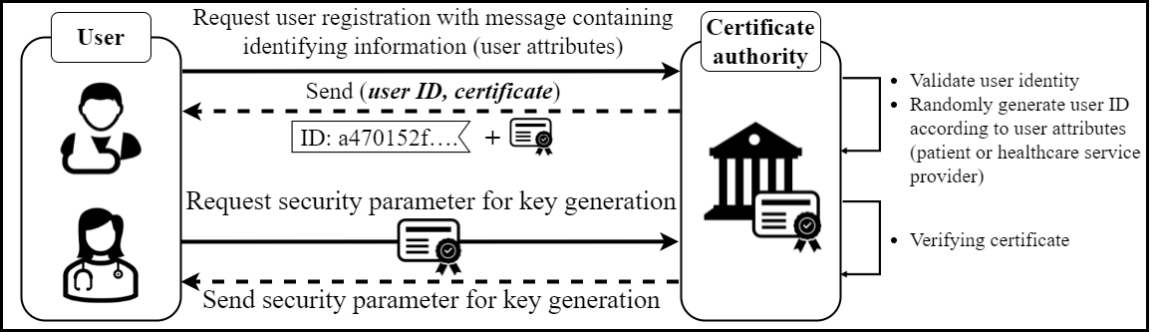
Initial phase: the user registration process to participate in the blockchain network.

Patients and physicians want to participate in the blockchain network to manage EMRs and securely share them with other users. Users participate in the blockchain network through the user registration process, which consists of an identity registration phase to register the user’s identity and an authentication phase to obtain security parameters to generate an encryption key. Figure 3 shows the user registration process for users to join the blockchain network. In the ID registration step, the user sends the ID registration request message that contains the user’s attributes to a CA. The user attributes identify whether the user is a health worker or a patient. The CA also uses the user attributes to identify the user’s ID. Once the identification is complete, if the user is legitimate, the CA classifies the user type based on the user attributes. Subsequently, depending on the user type, a user ID is generated and delivered to the user along with the certificate. Users who are judged to be not legitimate because their attributes are not validated will be denied user registration. A user who has successfully registered an account then sends a message, including the certificate, to the CA requesting security parameters to generate the encryption key needed for EMR sharing. If the certificate is valid, the CA provides the security parameters to the user. Thus, through the user registration step, users who join the blockchain network generate a public key and a private key using the security parameters received from the CA. First, the user selects a random decimal number corresponding to x ∈ Z^*^_q_. The selected *x* is set as the user’s secret key and is never shared. Next, users generate a public key for use in the network using their private key and the key generator. Users who can directly generate the encryption key that they use for EMR sharing can generate an encryption key each time an EMR is created, thus protecting the EMR with a different key each time. Because of the nature of an EMR that has been used for a long time, the encryption key must be maintained as is when data are encrypted [62]. However, this introduces security vulnerabilities, endangering EMR and patient privacy. To prevent this, in our system, users create the encryption key themselves, and the existing EMR uploaded to the IPFS is updated with data encrypted with the new key through a data version update. Users who successfully register an account and generate an encryption key can share medical data through the blockchain network.

#### Phase 1: EMR Upload—Block Generation

**Figure 4:**
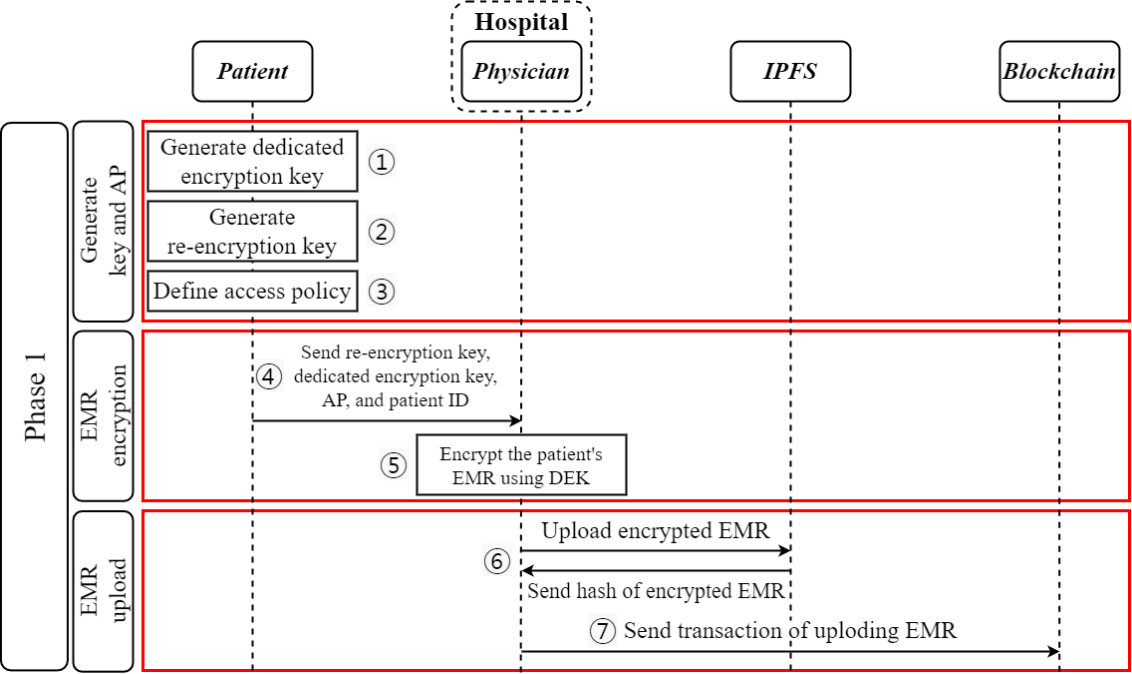
Phase 1: electronic medical record (EMR) upload flowchart of the proposed blockchain-based patient information exchange system. AP: access policy; DEK: dedicated encryption key; IPFS: InterPlanetary File System.

When a medical record is generated for treatment, the patient creates a dedicated encryption key to encrypt their EMR and a re-encryption key *RK_PDi_* to re-encrypt the encrypted EMR. The re-encryption key is generated using the patient’s private key and the physician’s public key. At the same time, the patient creates an AP that defines the level of users who can access the EMR. Next, the patient sends the dedicated encryption key *RK_PDi_*, the AP, and the user ID, which was created by the patient, to the physician who provided treatment. Upon receiving the message from the patient, the physician encrypts the patient’s EMR with the patient’s dedicated encryption key. The physician then uploads the encrypted EMR, *C_P_*, to the IPFS and receives the hash value, *hash(C_P_)*, of the data the IPFS has stored. Next, the physician submits the user ID related to the EMR, the minimum information required to distinguish *EMR_P_*, and *hash(C_P_)* to the medical institution to which they belong. Finally, the medical institution uses the information in the message to create a transaction and publish it on the blockchain. Figure 4 shows the EMR upload flow diagram of the proposed blockchain-based PIE system. The method of uploading EMR information to the blockchain follows algorithm 1 defined in Textbox 1.

**Algorithm 1: the electronic medical record upload.**

**Algorithm 1**

- Input: Secret key_Patient_, public key_Doctor_, dedicated encryption key, user ID_Patient_, hash(C_Patient_), and access policy
- Output: Re-encryption key, access policy, summary information from electronic medical record, hash value returned by the InterPlanetary File System, and transaction
  The patient selects a random security parameter value *r* to generate a dedicated encryption key for encrypting their electronic medical record.
  The patient uses their secret key *SK_P_* and the physician’s public key *PK_D_* to generate a re-encryption key *RK_P→Di_* for re-encrypting their *EMR_P_*.
  The patient generates an access policy that defines which users can access their electronic medical record.
  The patient transmits the dedicated encryption key, *RK_P→Di_*, access policy, and *UserID_P_* to the physician who treated them.
  The physician who receives the dedicated encryption key, RK_P→Di_, access policy, and *UserID_P_* from the patient encrypts the patient’s medical record EMR_P_ using the dedicated encryption key.
  The physician uploads the encrypted patient electronic medical record *C_P_* to the InterPlanetary File System and receives the *hash(C_p_)*, the hash value of the electronic medical record.
  The physician submits the patient’s ID, summary information from *EMR_P_*, and hash value to the hospital.
  The hospital uses the received information to create a transaction and uploads it to the blockchain network.

#### Phase 2: EMR Sharing

**Figure 5:**
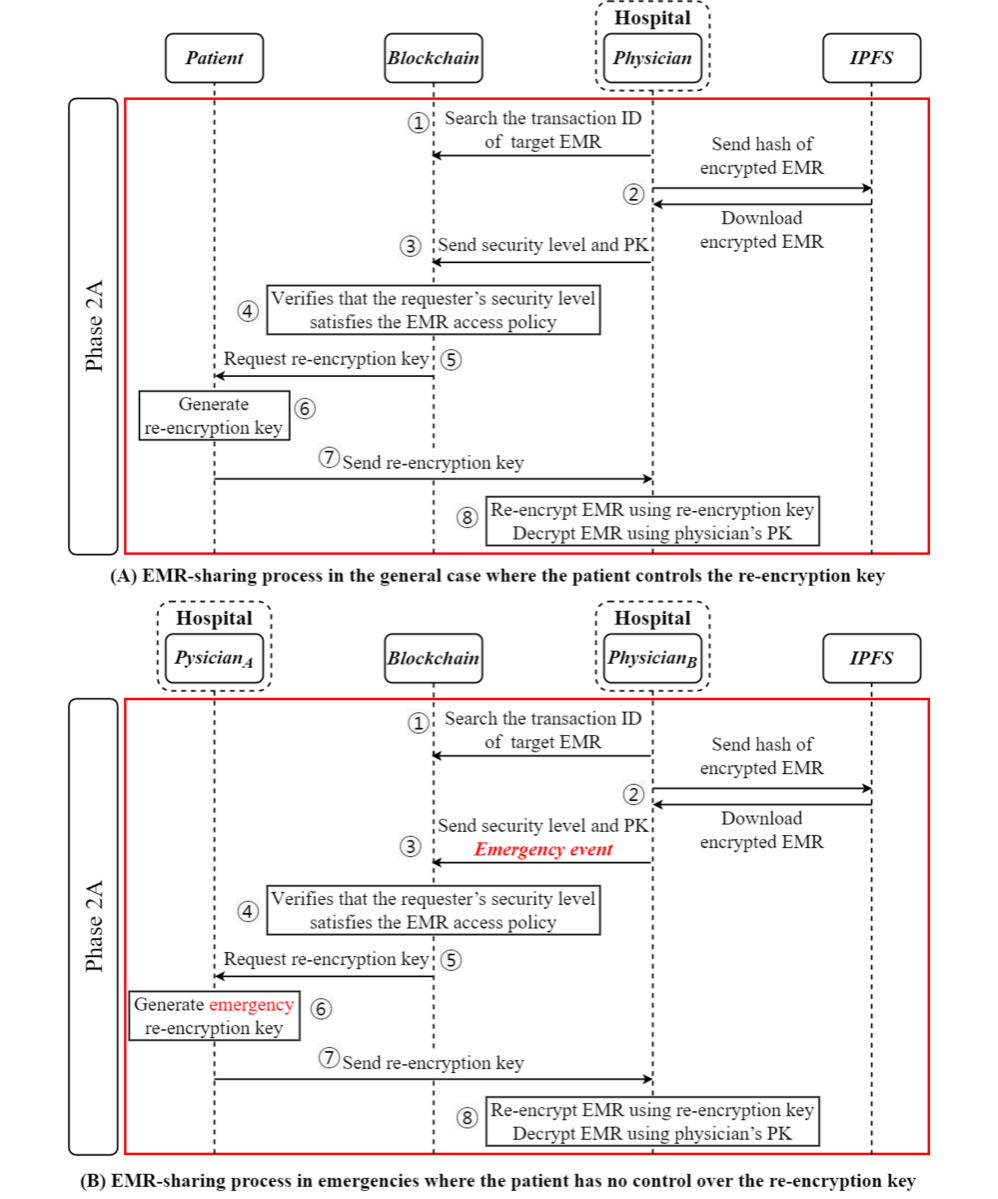
Phase 2: electronic medical record (EMR)–sharing process flowchart of the patient information exchange system. (A) EMR-sharing process in the general case where the patient controls the re-encryption key, (B) EMR-sharing process in emergencies where the patient has no control over the re-encryption key IPFS: InterPlanetary File System; PK: public key.

Users who want to share and use a particular EMR can search for it on the blockchain through a smart contract and request a re-encryption key. As the medical field is closely related to human life, the target and purpose regarding the EMR data must be legitimate. However, it would be difficult to find the desired EMR among countless data because it is hidden to protect the patient’s privacy. Therefore, in the proposed PIE system, smart contracts are applied so that patients and health care workers who are users of the blockchain network can perform a quick and accurate search for the data they want. Moreover, the efficiency of the EMR-sharing process has been improved by automating the process of requesting decryption rights after searching for an EMR. The requester uses summary information from the EMR and the user ID (the ID of the hospital that uploaded the data or the patient’s ID) to quickly search for a transaction containing the information of the desired EMR. Then, the requester downloads the encrypted EMR from the IPFS using the acquired transaction information. As the downloaded EMR is encrypted with a dedicated encryption key, it must be decrypted using the patient’s private key or re-encrypted using the re-encryption key before using it. However, because sharing the patient’s private key is very dangerous, the requester must send a message to the patient requesting a re-encryption key for re-encryption. The message requesting the re-encryption key goes through the user verification process of the access check contract. Initially, the user verification process checks whether the requester’s security level satisfies the patient’s EMR AP. When it is confirmed that the user has met the required security level, a message requesting a re-encryption key is sent to the patient, the owner of the EMR. Upon receiving the message requesting the re-encryption key, the patient sends *RK_PRequester_* to the requester. If a patient cannot issue a re-encryption key because they have been incapacitated by a serious illness such as acute stroke, the physician participating in the EMR generation can temporarily issue a re-encryption key according to the emergency event procedure. The requester who receives *RK_PRequester_* can re-encrypt *C_P_* and then decrypt with their private key. The EMR-sharing procedure is performed in the order specified in Textbox 2. The EMR-sharing flowchart of the proposed blockchain-based PIE system is illustrated in Figure 5. The biggest advantage of using PRE technology for medical data security is that users can decrypt a downloaded EMR encrypted with their key without the patient’s private key. Therefore, it minimizes the threat of leakage of the patient’s private key and information. Moreover, the proposed re-encryption technology–based medical data encryption method can satisfy the medical field’s requirements for sharing medical data while protecting the medical data from other eyes when sharing the EMR. Figure 5 shows the EMR-sharing procedure in the proposed PIE system.

**Algorithm 2: electronic medical record sharing.**

**Algorithm 2**

- Input: Summary information from the electronic medical record, user ID, and user’s security level
- Output: Re-encrypted electronic medical record *C_d_* and *RK_P→Requester_*
  The physician executes a smart contract for electronic medical record retrieval to find a transaction containing the information of the desired electronic medical record.
  The smart contract uses the user ID or the electronic medical record’s summarized information to find a transaction containing the desired information and returns it.
  The physician who receives the transaction information downloads the encrypted electronic medical record *C_P_* from the InterPlanetary File System using the hash value contained in the transaction.
  The physician executes the re-encryption key request smart contract to request the key *RK_EMRP_* for re-encryption from the patient who is the owner of the encrypted electronic medical record.
  The smart contract for requesting the re-encryption key performs a user authentication step to verify that the security level submitted by the user who requested the message transmission satisfies the access policy set by the patient.
  If the security level of the user requesting the re-encryption key satisfies the access policy, the smart contract sends a message requesting the re-encryption key to the patient (if the user’s security level does not meet the criteria, the request is denied).
  The patient who receives the message requesting the re-encryption key generates *RK_P→Requester_* using the requester’s public key and send it to the requester.
  The physician receiving *RK_P→Requester_* uses it to re-encrypt *C_P_* into *C_D_*.
  The physician uses their private key to decrypt *C_D_* to obtain the original *EMR_P_*.

## Results

### Simulation Design

A simulation was designed to verify that the proposed blockchain-based medical system sufficiently reflects the medical field’s requirements and enables safe data sharing. We simulated the process of sharing encrypted EMRs over a blockchain network. In the EMR-sharing process of uploading and downloading EMRs over the network, we checked the effect of the time taken to process the data and the size of the data shared on the system’s performance. Moreover, the performance of the proposed smart contract–based re-encryption key–sharing method was verified by measuring the execution time of the smart contract. This paper does not cover improvements to the consensus process performed on the blockchain network; therefore, the improvements were not evaluated.

The test environment was designed based on the data-sharing process defined in the *Methods* section. The simulation consisted of 2 physicians, a patient, a medical consortium, and a public IPFS network participating in the blockchain network as the minimum unit for sharing an EMR. Entities on the network were classified into two categories: a host operating a blockchain network and a guest operating on the network. All processes in the proposed system can be viewed as interactions among guests. In the test environment, there was a consortium to which many hospitals belonged, and each hospital had a Flask server with a different port. The code of the software development kit used to implement the blockchain network is Node.js. The host computer was a PC running Windows 10 Pro 64x (Microsoft Corp) in a wired environment, with 32 GB memory and Intel Core i7-10700K central processing unit at 3.80 GHz. The guest computer was a PC running Ubuntu 18.0.4 64x (Canonical) in a wired environment, with 10 GB memory and Intel Core i7-10700K central processing unit at 3.80 GHz. The communication rates considered were the download and upload rates in megabits per second: host upload and download rates and guest upload and download rates.

The simulation was performed on 1 PC to directly compare the host’s and guest’s processing times. Blockchain implementation was performed using Hyperledger 2.3.1 [63]; Apache CouchDB [64] was the state database. The network consisted of 4 orderer nodes, 3 organizations, 2 peer nodes for each organization, and 1 channel. The chain code for smart contracts used Go. Table 7 shows the parameters used for the proposed PIE system simulation.

**Table 7:** Simulation parameters.

Parameters	Values
Data size	0.4 kB, 1 MB, 10 MB, 100 MB, and 1 GB
Data type	CSV (text) and DICOM^a^ (images and videos)
Number of orderer nodes	4
Number of organizations	3
Number of peer nodes	6
Number of channels	1
Data rate	100 Mbps
Block size	1 MB
Block timeout	2 seconds
Database	Apache CouchDB

^a^DICOM: Digital Imaging and Communications in Medicine.

### Simulation Results

To evaluate the performance of the proposed PIE system, we measured the time required for the EMR-sharing process and the execution time of the smart contract for re-encryption key sharing. The EMR-sharing process was divided into upload and download processes, and the time taken to perform each process was measured. The execution time of the EMR upload process was defined as the time taken to upload the EMR to the IPFS and post the returned EMR hash value to the blockchain. The execution time of the EMR download process was a measure of how long it took users to download the EMR over the IPFS. Considering the characteristics of an EMR that supports various types of data, the simulation was performed using various data, ranging from text format (0.4 kB) to medical images (1 GB). The simulation measured only the time required in the communication process for exchanging data among users and did not consider the impact on the process of the data encryption and decryption operations. To objectively evaluate the performance of the proposed system, we performed a comparative analysis with existing blockchain-based medical information exchange systems. Simulations were performed for three types of systems (an on-chain–based system designed for cryptocurrency, an Ethereum-based system using the IPFS, and a PIE system); the results of the simulations for the EMR upload process are shown in Multimedia Appendix 2 [47,48].

Through the EMR upload simulation it was confirmed that the larger the data to be uploaded, the longer it takes; the larger the amount of data to be uploaded, the higher the required data rate. As a result, the processing time increased dramatically for data that exceeded the acceptable data rate (100 Mbps) in the simulation environment. In an on-chain–based system that stores data in the original form in blocks, the size of data that can be uploaded is limited to 1 MB, which is the maximum size of the block; therefore, there is no simulation result for data beyond that size. Most of the time taken to upload an EMR was when a query request needed to be made to the blockchain network, which took an average of 2.1 (SD 0.0343) seconds. The actual time taken to upload an EMR to the IPFS increased depending on the size of the data, but it was very short. A graph of the time it takes to upload EMR to IPFS can be found in Multimedia Appendix 3. The time required to upload data that ranged from 0.4 kB to 100 MB was relatively short compared with the query request time; therefore, it did not significantly affect the overall EMR upload time. Again, when uploading data that ranged from 0.4 kB to 100 MB, the overall EMR upload time was comparable with the query request time (average of 2.1 seconds, SD value = 0.2947). However, when uploading >500 MB of data, the time taken to upload the EMR to the IPFS was longer than the query request time, which affected the overall EMR upload time. Uploading 500 MB and 1 GB of data took an average of 4.5 (SD 0.1329) seconds and 5.7 (SD 0.21) seconds, respectively, for the Ethereum-based system and the proposed PIE system. The PIE system and the Ethereum-based system showed similar performance in that the EMR was distributed and shared using the IPFS. However, in the Ethereum-based system, there is a problem: to decrypt the shared medical data, the patient’s private key needs to be shared or the patient needs to directly decrypt the shared medical data. This gives rise to a fatal security problem: the patient’s private key and EMR can be leaked directly to others. In contrast, the PIE system prevents the leakage of the patient’s private key and EMR by using a re-encryption scheme and enables users who have shared their EMRs to decrypt them smoothly, providing high security along with the same performance as that of the Ethereum-based system. Uploading the actual EMR to the IPFS and sharing it through a decentralized technique has 3 important implications in a blockchain-based medical data system. First, medical data can be shared without capacity limitation through a peer-to-peer network. This advantage can thus alleviate the problem of low processing efficiency and data scalability because of the blockchain’s limited block capacity. Second, by storing the hash value, which is the unique address value of the data, it is possible to reduce the blockchain’s storage burden and solve the EMR reduced redundancy storage problem. When sharing an EMR, the PIE system reduces the burden on the nodes and allows data to be shared faster. The distributed data-sharing method using the IPFS determines the performance according to the number of users sharing data, showing higher performance as the number of users increases. Therefore, EMR sharing using the distributed data-sharing method is effective for the medical system because it can share data faster while reducing the burden on the node. However, the data-sharing method using the IPFS has a problem in that there must be at least one node that stores the data to be shared in the IPFS network. If the data are not stored (pinned) on the IPFS network, the shared system can fail. To prevent this, the hospital that created the EMR needs countermeasures such as storing data in a local database in preparation for the worst-case scenario after uploading.

The simulation results for downloading the EMR posted on the blockchain are presented in Multimedia Appendix 4 [47,48]. The simulations we conducted used various data sizes ranging from 0.4 kB to 1 GB. The IPFS used in the simulations is an open network, with a variable number of users participating in data-sharing operations. Therefore, even with a blockchain system using the same IPFS, differences in performance may occur depending on the number of users participating in the network. The Ethereum-based blockchain system [48] and the PIE system we propose use the IPFS to alleviate the blockchain’s scalability problem. However, there is a big difference between the 2 models in the pieces of information they store. In the model proposed in the study by Wu and Du [48], only the detailed information of the EMR is stored, whereas in the PIE system we propose, the original EMR is stored as is. Storing the original data of the EMR generated in the medical process can ensure the integrity of medical data and increase its usability in medical systems. The simulation results show the difference in performance between storing only the detailed information of the EMR and storing the original EMR as is. Even considering that the performance of the IPFS may fluctuate depending on the number of participating users, it takes less time to download the original EMR than it takes to download the details of the EMR. These results mean that the proposed PIE system provides higher scalability in that it can provide the original EMR to users more quickly. The average time taken to download 1 MB of medical data encrypted using the unique address value of the medical data uploaded to the IPFS in the proposed PIE system is 0.01014 (SD 0.0028) seconds. This is approximately 5.5 times faster than the average download time of 0.0562 (SD 0.0052) seconds taken by the on-chain–based blockchain model without the IPFS. Existing blockchain systems that publish and share data without using the IPFS increase the burden and have limitations in scalability as the size of the data to be shared increases. However, there is no limit to the size of the EMR posted on the blockchain in the proposed method, ensuring high scalability and higher processing performance than that of existing systems. Therefore, for medical systems that need to share medical data, it is more effective to use the distributed data-sharing method.

We performed a simulation of the smart contract–based re-encryption key–sharing process. The re-encryption key–sharing process verifies the user requesting the re-encryption key to use the patient’s EMR and passes the re-encryption key to the user. The user who receives the re-encryption key performs the re-encryption process and finally decrypts the encrypted EMR using their private key to use the patient’s EMR. A graph of the smart contract simulation results for re-encryption key sharing is in Multimedia Appendix 5. The simulation was performed with 4000 epochs, and the quarterly smart contract execution time and average time required are presented. As the re-encryption process and decryption process are performed by the user alone, they are not included in the smart contract’s execution time for sharing the re-encryption key. The average time taken to verify a user requesting a re-encryption key to use the patient’s EMR and grant decryption rights to an authorized user is 3.3543 (SD 0.4959) seconds. The data security process using the re-encryption method effectively protects medical data; in addition, it can provide convenience to users while protecting data from various security threats. The simulation results confirmed that the proposed PIE system provides higher scalability and stronger security performance than existing blockchain-based medical systems.

### Security Analysis

In this section, we will check how the proposed PIE system effectively responds to security threats and analyze whether it is possible to share secure medical data using the proposed PIE system.

#### Strong Privacy Preservation

When medical data are shared using a network, an external attacker can obtain the medical data through a sniffing or eavesdropping attack. If medical data are leaked, the patient’s privacy in the EMR is also exposed. In the proposed PIE system, medical data are encrypted using the dedicated encryption key for the safe sharing of medical data. As encrypted medical data can only be decrypted by the patient or by a user approved by the patient, the information in the data is not exposed even if the data are stolen. By granting data decryption authority using the PRE technique, the user approved by the patient can decrypt the data using their private key during the data decryption step. The proposed EMR-sharing method prevents the leakage of private information during the EMR-sharing process and ensures safety by eliminating the private key–exchange process for data decryption. If an internal attacker attempts unauthorized access to the patient’s information, in our proposed system, *Smart contract_RKrequest_* verifies the requester’s security level and accepts or declines the request depending on the AP set by the patient. The access control scheme using a smart contract can protect the patient’s privacy from internal threats.

#### Data Integrity

The internal attacker can perform forgery attacks by accessing the medical data that medical institutions manage independently. If the original data stored at a medical institution are damaged, it is difficult to recover the data; moreover, it is also significantly challenging to determine whether the data have been forged or altered. These attacks can be effectively prevented by storing and managing EMR-related information such as the hash value of medical data, publicly available medical information, and hospital ID in the blockchain. As the EMR information recorded on the blockchain contains the information at the time of creation, it is easy to check whether the data are damaged. If the data are damaged, they can be quickly restored using the distributed data-sharing method. For an attacker to forge the data stored in the blockchain, they must possess the mighty hash computation power of more than 50% of the entire network and create new blocks faster than other honest nodes propagate them to the network. As meeting the necessary conditions to forge blockchain data is challenging, attackers cannot delete or modify data. Therefore, using a blockchain-based medical system ensures medical data integrity and reliability, thereby enabling safe medical data management and sharing.

#### Network Security

The external attacker can perform denial-of-service attacks on the PIE system. As a result, the system’s operation becomes abnormal and it produces unexpected outputs. The system we propose is directly or indirectly related to patients’ lives; therefore, high availability is important. Hence, we use distributed systems such as a medical consortium blockchain and the IPFS. If the attacker breaks down the sharing system, patients cannot share their medical data and physicians or health care providers cannot obtain the required information. However, in the proposed sharing scheme based on blockchain, if the attacker makes a few of the blockchain nodes unavailable, the other nodes can provide the needed services.

## Discussion

### Principal Findings

The study’s principal findings concern implementing integrated management of fragmented EMRs, preventing leakage of personal information of patients during the EMR-sharing process, and establishing a patient-centered medical data system by granting decryption authority, as outlined in the following list:

1. We designed a blockchain-based PIE medical system that effectively manages and shares medical data. EMRs generated by different medical institutions are managed through a blockchain network to prevent the fragmentation of medical data. Moreover, through the PIE system, duplicate EMRs can be avoided, reducing the cost and wastage of storage space.
2. The PIE system encrypts the patient’s medical data and uploads and shares the encrypted EMR and data identification parameters to the network with minimal medical information. Thus, the proposed method fundamentally overcomes the problem of possible leakage of personal data when the data are posted on the blockchain for sharing with other network members. Therefore, privacy preservation required in a system handling sensitive EMR information is guaranteed, making safe EMR management and sharing possible.
3. Our system reinforces the patient’s role in the medical system by allowing them to grant decryption rights to their data to other users using re-encryption techniques. If other users (eg, physicians or researchers) wish to use a patient’s EMR data, they must obtain a re-encryption key and re-encrypt the EMR data. Building a patient-centered medical data system differs from the existing hospital-centered medical data system in that the patient’s role is reinforced in our system.

### Limitations and Future Work

Blockchain-based medical systems receive considerable attention as next-generation medical systems that will replace existing medical data management systems, and numerous researchers are conducting various studies. However, blockchain-based medical systems’ technological maturity remains at the prototype level. Moreover, as the medical data formats used by different countries or institutions vary considerably, it is challenging to share the medical data. Consequently, research into standardized medical data formats such as the Health Level 7 Fast Healthcare Interoperability Resources [65] is required. To successfully create the next-generation medical environment through a blockchain-based medical system, various and complex issues such as backlash from the medical field, legal ramifications related to medical care, technical limitations, and data standards must be addressed [66,67]. We aim to conduct research on public medical data systems that enable safe sharing of medical data in public networks as well as data security techniques so that EMRs can be used in more diverse fields in the future [68].

### Conclusions

This paper presented the PIE system based on a consortium blockchain that allows patients to manage their medical data. The PIE system can securely manage and share EMRs by overcoming the existing blockchain-based medical system’s problems. The PIE system uses a distributed data-sharing method and lightweight transaction structure to solve scalability and privacy issues, a chronic problem of blockchain-based medical systems. By rapidly sharing large-capacity data such as medical images using a distributed data-sharing method, the issues of low processing speed and block sizes of existing blockchains are addressed. Lightweight transactions can store more information in blocks because they contain only minimal information, such as the encrypted EMR metadata and EMR summary information. The vast amount of medical data generated daily is effectively processed and managed using a lightweight transaction structure. The re-encryption–based data encryption method is used to resolve the problem of leakage of data and personal information when sharing EMRs. Even if the EMR encrypted with the dedicated encryption key is leaked during the sharing process, it cannot be decrypted; therefore, it is safe from the threat of leakage. Honest users wishing to use the patient’s data can re-encrypt the EMR by obtaining a re-encryption key from the patient. The EMR-sharing process was performed using smart contracts. Security level–based access control was performed using smart contracts to prevent unauthorized users from using medical data, and re-encryption keys were delivered only to authorized users. As a result, the proposed blockchain-based medical system provides improved security and scalability, enabling efficient and safe medical data sharing.

## Appendix

Multimedia Appendix 1 Proxy Re-encryption based EMR Encryption & Decryption.

Multimedia Appendix 2 Average data upload time by electronic medical record size.

Multimedia Appendix 3 Average data upload time to the InterPlanetary File System by electronic medical record.

Multimedia Appendix 4 Average data download time by electronic medical record size.

Multimedia Appendix 5 Smart contract execution time for re-encryption key sharing.

## Abbreviations

AP: access policy
CA: certificate authority
EMR: electronic medical record
IPFS: InterPlanetary File System
ONC: Office of the National Coordinator for Health Information Technology
PIE: patient information exchange
PRE: proxy re-encryption
TTP: trusted third party
