# Supplementary material for: Privacy Preservation in Patient Information Exchange Systems Based on Blockchain: System Design Study
Source: J Med Internet Res. 2022 Mar 22;24(3):e29108. doi: 10.2196/29108 (PMC8984831; doi:10.2196/29108)
Supplement: Multimedia Appendix 1 [file jmir_v24i3e29108_app1.docx]

# Multimedia Appendix 1

## Proxy Re-encryption based EMR Encryption & Decryption

The entire EMR sharing scenario is shown as Figure 1. In the following sections, we explain the processes of our sharing scenario that focus on EMR encryption, re-encryption, and decryption in the following sections.


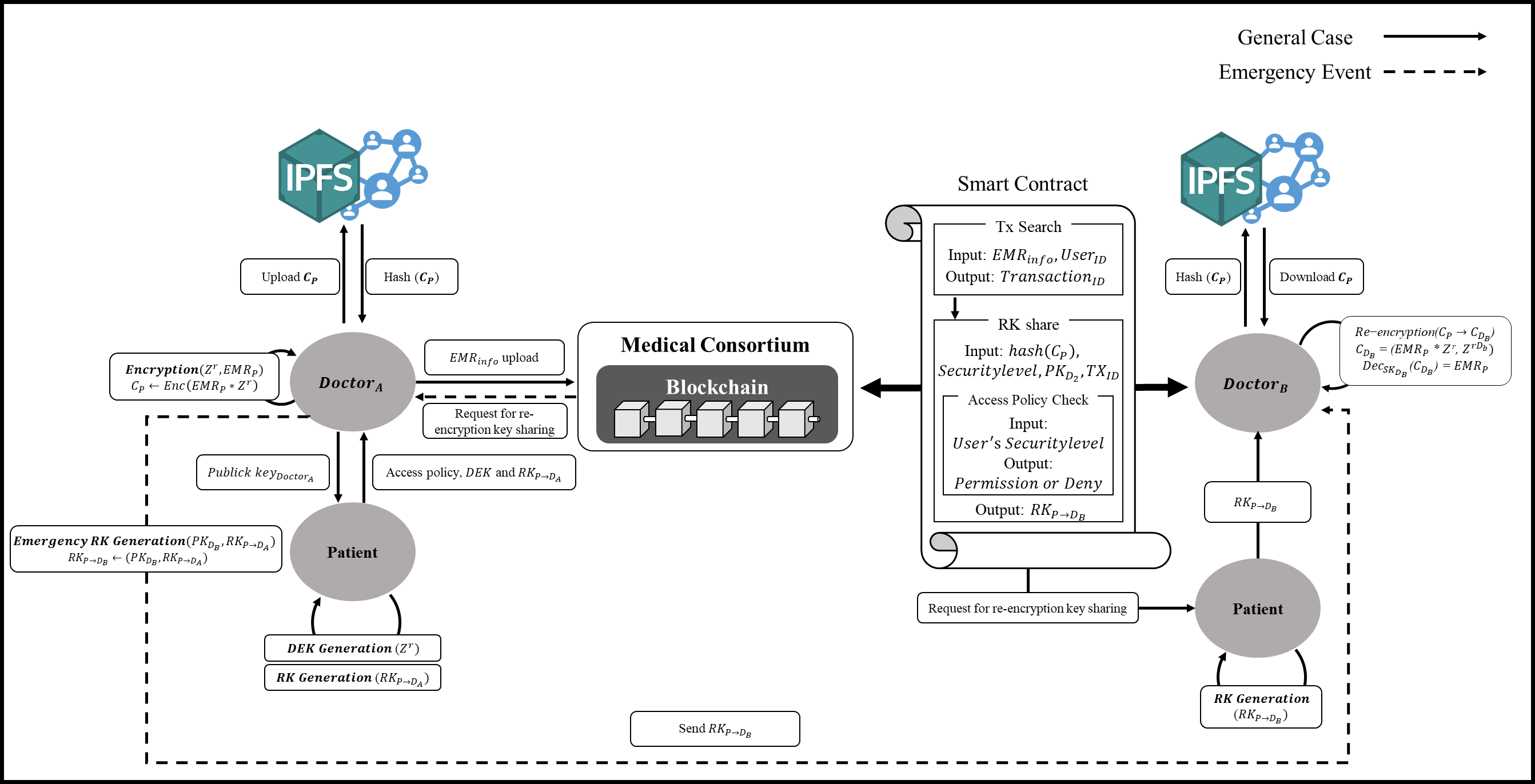


Figure 1. The EMR sharing scenario in the proposed system

### System Initialization

For the EMR encryption and decryption based on proxy re-encryption, we define the security parameter as Textbox 1.

Textbox 1. The security parameter used for EMR encryption in the proposed medical system

| - $g$: generator of $\mathbb{G}_{1}$ - $q$: large prime order - $\mathbb{G}$**:** cyclic groups of prime order *q* - $e$: bilinear map of the form $\mathbb{G}_{1}\times\mathbb{G}_{1}\to\mathbb{G}_{2}$ where $\mathbb{G}_{1}=<g>$. $e$ must be efficiently computable. Also, $e$ must be non-degenerate; that is,$<e(g,g)>=\mathbb{G}_{2}$. If for all $g\in\mathbb{G}_{1},$*a, b* ∈ $\mathbb{Z}_{q}$, that $Z=e\left( g,g \right)$, $e\left( g^{a},g^{b} \right)=e\left( g,g \right)^{ab}$. - *SK**: Private key of *** - *PK**: Public key of *** |
| --- |

### EMR encryption

For the storing and uploading EMR securely, the patient encrypts the EMR data. When an EMR data is generated by treatment, the treated patient encrypts the EMR data using the patient’s private key ($SK_{p}=p)$ and randomly selected number $r$. The algorithm of EMR encryption is defined in Algorithm 1.

Algorithm 1. EMR encryption

| Data encryption |
| --- |
| Input: *Private key* of the patient ${SK}_{p}$, $EMR_{P}$  Output: Encrypted $EMR_{P}$: $C_{P}$ |
| **Function** Encryption ($SK_{p}, EMR_{P}$)  **if** EMR is generated through treatment **then**  Select random $r$  EMR encryption key $Z^{r}=e(g,g^{r})$  $C_{P}\leftarrow(Z^{r}*EMR_{p}$*,* $g^{rp})$  **return** $C_{P}$  **end function** |

After the EMR encryption, the patient uploads the encrypted EMR ($C_{P}$) to the blockchain. The upload process described as Algorithm 2.

Algorithm 2. Data upload

| Data Upload |
| --- |
| Input:$UserID\left( P,D_{i} \right)$, *hash*$(C_{P}\boldsymbol{)}$, *Access Policy*, $Sign_{D_{i}}$, $Medical_{info}$  Output: *Transaction* about$C_{P}$ |
| **function** Data Upload $(UserID\left( P,D_{i} \right)\boldsymbol{,}$*hash*$(C_{P}\boldsymbol{)}$, *Access Policy*, $Sign_{D_{i}}$, $Medical_{info}\boldsymbol{)}$  **if** the hospitals receive EMR-related information from doctors, **then**  $hash\left( C_{P} \right)\leftarrow$Upload $C_{P}$ to IPFS system  A transaction containing the *hash*$\left( C_{P} \right)\boldsymbol{,}$ *User ID, Timestamp, simple medical information, access policy, and doctor's signature* is created.  $Transaction=\left\{ \begin{aligned} UserID\left( P,D_{\boldsymbol{i}} \right), Timestamp, Medical_{info}\left( EMR_{P} \right), \\ Metadata\left( hash\left( C_{P} \right) \right),A{ccessPolicy}_{EMR_{P}}, Sign_{D_{\boldsymbol{i}}} \end{aligned} \right\}$  Send transactions to the blockchain network.  **end if**  **end function** |

### EMR re-encryption

When a requester desires to get the EMR data of the patient, the patient generates the re-encryption key using his private key and the requester’s public key. Assuming the patient’s private key ($SK_{P})$ is $p$ and the requester’s public key ($PK_{R}$) is $g^{q}$, the patients can generate the re-encryption key by the equation (1). Algorithm 3 shows that the general case of generating the re-encryption key.

$Re Encryption Key Genenration: {RK}_{P\to R}=\left( PK_{R} \right)^{\frac{1}{SK_{p}}}=\left( g^{SK_{R}} \right)^{\frac{1}{p}}=g^{\frac{q}{p}}$ ( 1 )

Algorithm 3. Re-encryption key generation

| Re-encryption key generation |
| --- |
| Input: A's *Secret key* and B's *Public key*  Output: $Re$-$Encryption Key({RK}_{A\to B}$) |
| **function** RK Generation ($PK_{B}, SK_{A}$)  **if** EMR is generated through treatment **then**  Generate a Re-encryption key ${RK}_{A\to B}$  ${RK}_{A\to B}\leftarrow RK\_Gen\left( PK_{B}, SK_{A} \right)=\left( g^{b} \right)^{\frac{1}{a}}=g^{\frac{b}{a}}$  **else**  Do nothing  **return** ${RK}_{A\to B}$  **end function** |

After generation of the re-encryption key, the patient sends the re-encryption key to the requester. Obtaining the re-encryption key, the requester re-encrypts the encrypted EMR data using the received re-encryption key ($RK_{P\to R}$) as the equation (2). The detailed algorithm is shown in Textbox 10.

Using the patient’s private key ($SK_{p}$) and the doctor’s public key ($PK_{D}=g^{d}$), the patient can generate the re-encryption key for doctor ($RK_{P\to D})$. After generating the re-encryption key, the patient encrypts the EMR data. The detail of EMR encryption algorithm is defined in Algorithm 4.

$Re encrypt C_{P}=\left( Z^{r}*EMR_{P}, e\left( g^{rp},g^{\frac{q}{p}} \right) \right)=\left( Z^{r}*EMR_{P}, Z^{rq} \right)$ ( 2 )

Algorithm 4. Re-encryption

| Re-Encryption |
| --- |
| Input: $Encrypted EMR\left( C_{P} \right)$, $Re$-$encryption Key(RK_{P\to R})$  Output: $Re$-$encrypted EMR$($C_{Requester}$) |
| **function** Re-encryption ($C_{P}, RK_{P\to R}$)  **if** Re-encryption is performed for the encrypted EMR using the re-encryption key,**then**  Re-encrypt the $C_{P}$using the re-encryption key $RK_{P\to R}$.  $C_{R}\leftarrow\left( Z^{r}*EMR_{P}, e\left( g^{rp}, RK_{P\to R} \right) \right)$  $\boldsymbol{=}\left( Z^{r}*EMR_{P}, e\left( g^{rp}, g^{\frac{q}{p}} \right) \right) =(Z^{r}*m, Z^{rq}$)  **else**  Do nothing  **end if**  **return** $C_{R}$  **end function** |

### EMR decryption

To derive the EMR data from $C_{P}$ or $C_{R}$, the patient or requester can operate EMR decryption process. The detail of the process is described in the equation (3), (4) and Algorithm 5.

$Decryption by the requester: EMR_{P}=\frac{Z^{r}*EMR_{P}}{\left( Z^{rq} \right)^{1/q}}=\frac{Z^{r}*EMR_{P}}{Z^{r}}=EMR_{p}$ ( 3 )

$Decryption by the patient:EMR_{p}=\frac{Z^{r}*EMR_{P}}{\left( Z^{rp} \right)^{1/p}}=\frac{Z^{r}*EMR_{P}}{Z^{r}}=EMR_{p}$ ( 4 )

Algorithm 5. Decryption

| Decryption |
| --- |
| Input: $Encrypted EMR_{P}$($C_{A}) and S$*ecret key* ($SK_{A}$)  Output: $Original EMR_{P}$ |
| **function** Decryption ($C_{A},SK_{A}$)  **if** $Encrypted EMR (C_{A})$is obtained,**then**  ${EMR}_{P}\leftarrow Decryption\left( C_{A}, SK_{A} \right)=\frac{Z^{r}*EMR_{P}}{e\left( g,g^{ra} \right)^{1/a}}=\frac{Z^{r}*EMR_{P}}{Z^{r}}$  **else**  Do nothing  **end if**  **return** $EMR_{P}$  **end function** |
