# Supplementary figures and images for: Privacy Preservation in Patient Information Exchange Systems Based on Blockchain: System Design Study
Source: J Med Internet Res. 2022 Mar 22;24(3):e29108. doi: 10.2196/29108 (PMC8984831; doi:10.2196/29108)

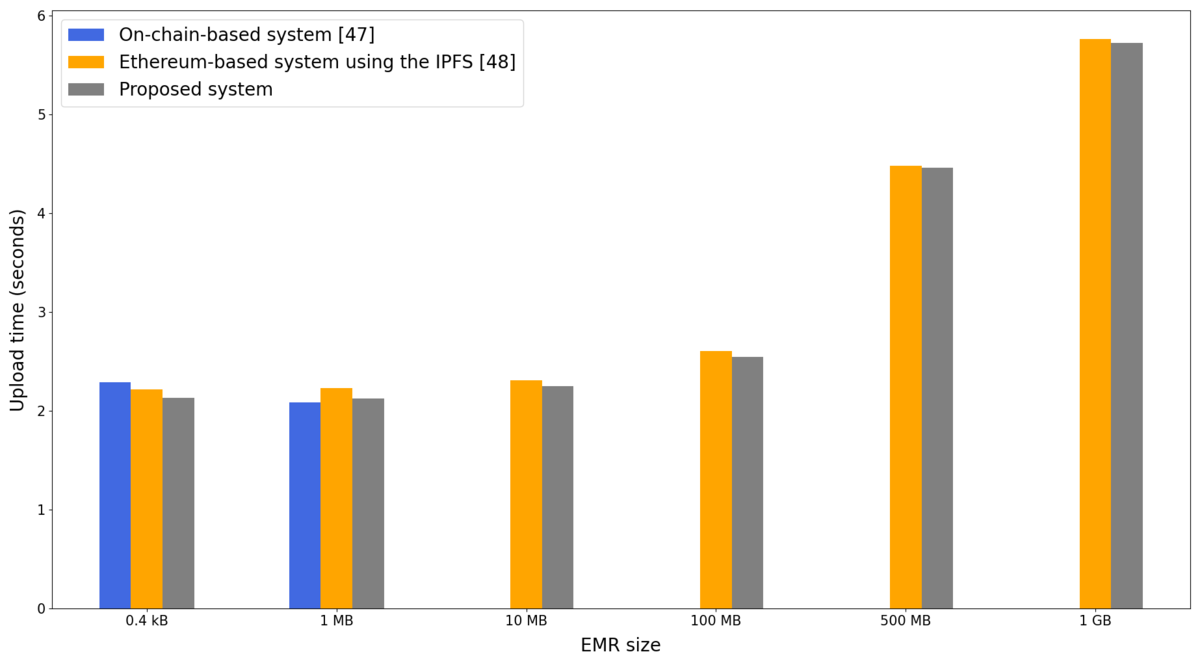

Supplement: Multimedia Appendix 2 [file jmir_v24i3e29108_app2.png]

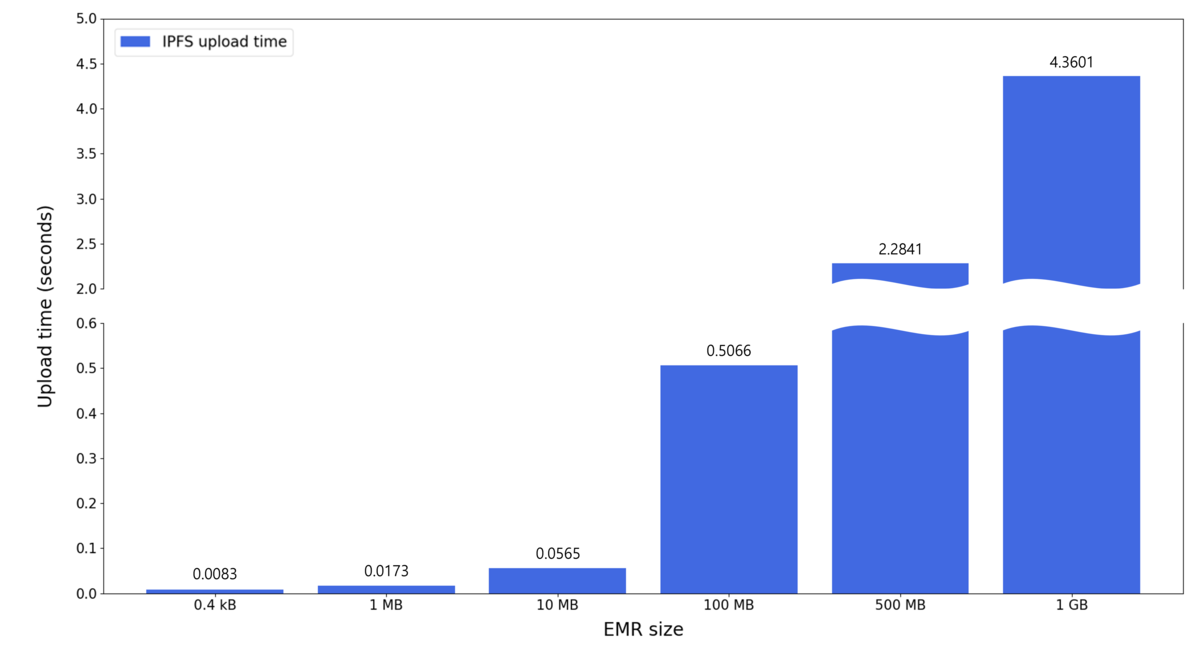

Supplement: Multimedia Appendix 3 [file jmir_v24i3e29108_app3.png]

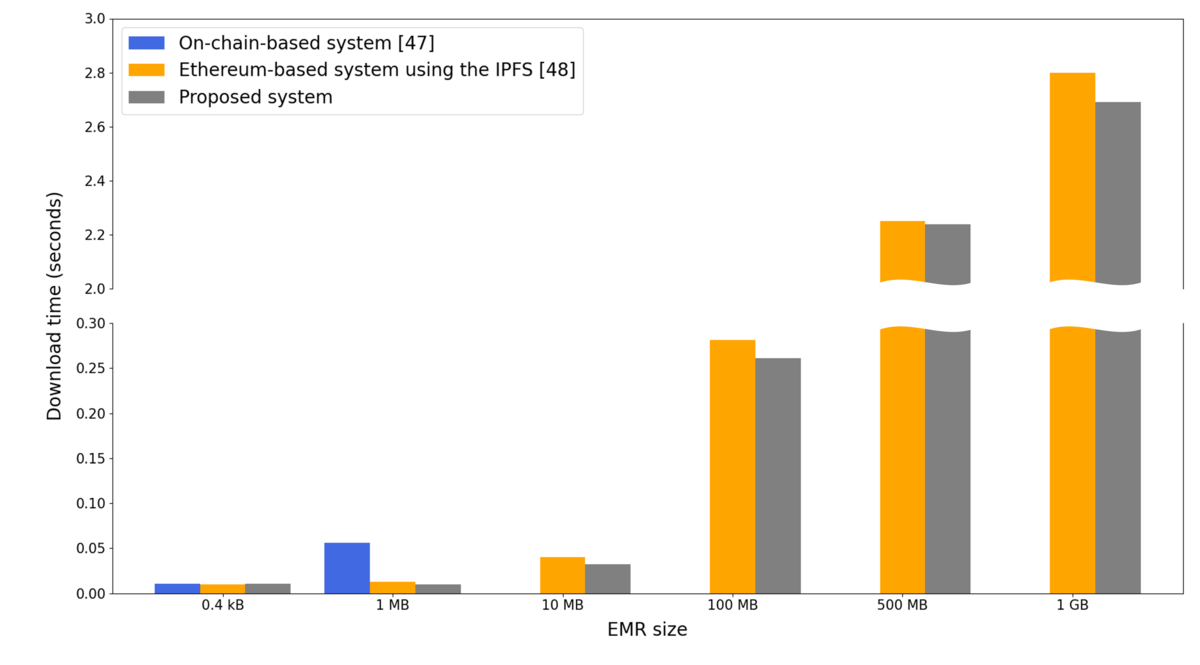

Supplement: Multimedia Appendix 4 [file jmir_v24i3e29108_app4.png]

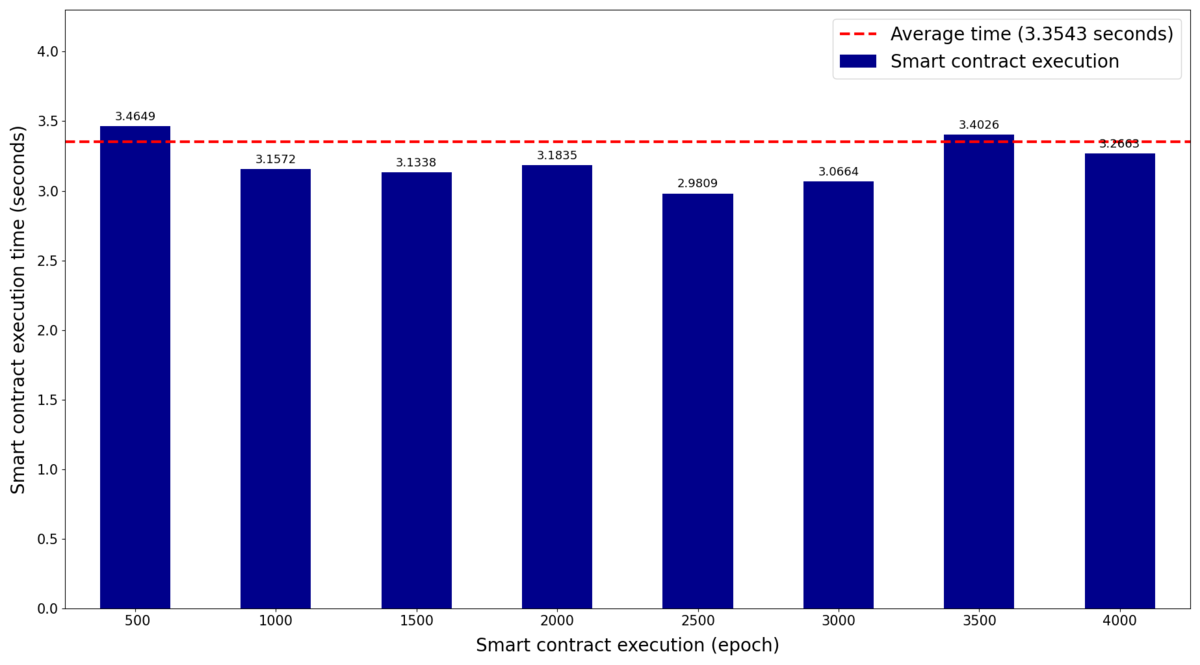

Supplement: Multimedia Appendix 5 [file jmir_v24i3e29108_app5.png]
